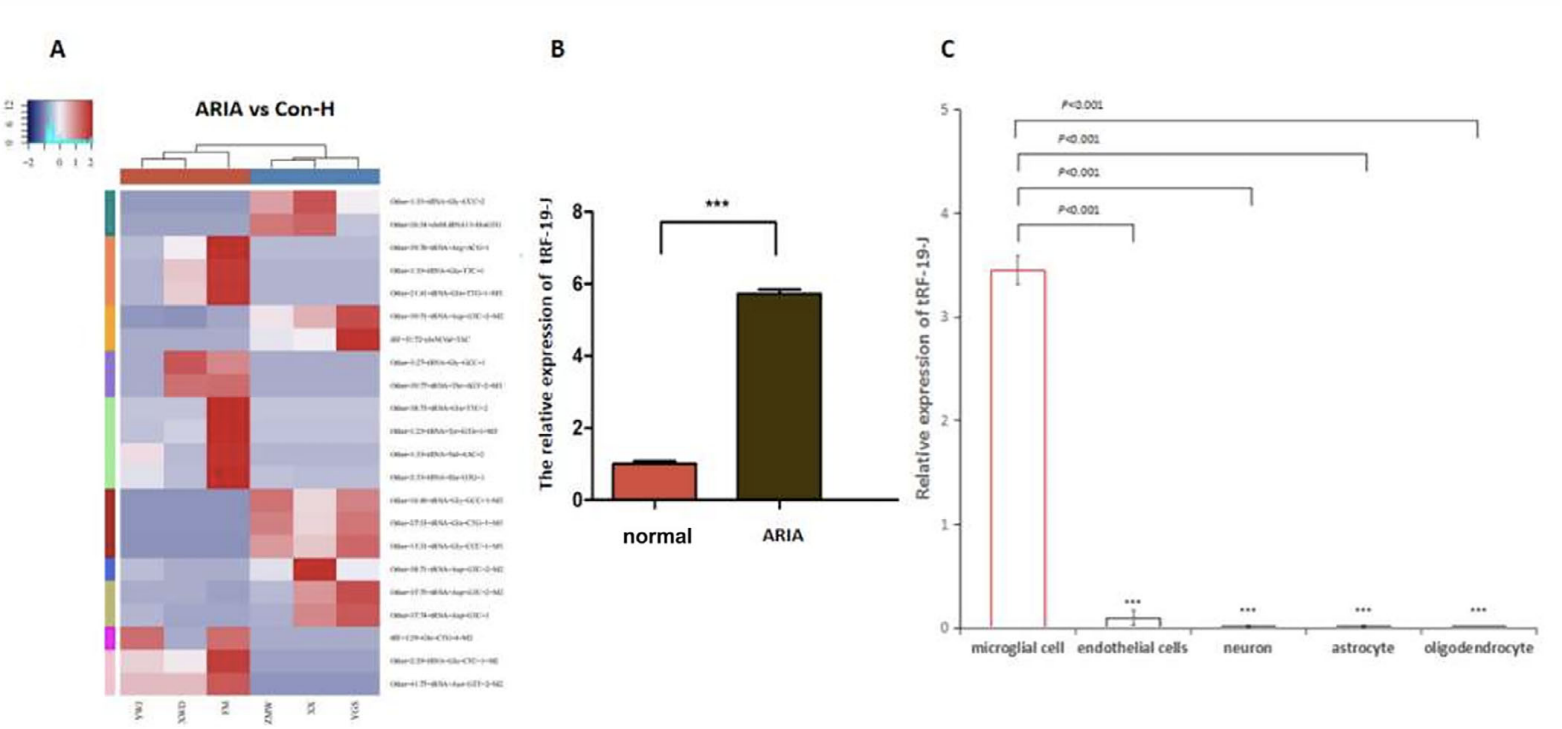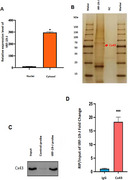# Upregulation of tRF‐19‐J in Anti‐Aβ Therapy‐Related ARIA Among Alzheimer's Disease Patients and Its Role in Promoting Ubiquitin‐Mediated Degradation of Connexin 43 in Brain Endothelial Cells

**DOI:** 10.1002/alz70861_108391

**Published:** 2025-12-23

**Authors:** Min Fang

**Affiliations:** ^1^ St. Luke’s Hospital, Shanghai, Shanghai China

## Abstract

**Background:**

Amyloid‐related imaging abnormalities (ARIA) are serious complications commonly observed during anti‐Aβ monoclonal antibody therapy for Alzheimer's disease (AD). Disruption of blood‐brain barrier (BBB) permeability is considered a key mechanism in ARIA development. Recent studies have shown that tRNA‐derived fragments (tRFs) are aberrantly expressed in AD and may be involved in regulating Aβ neurotoxicity and glial cell activation. However, whether tRFs participate in the modulation of BBB permeability remains largely unexplored.

**Method:**

The study included AD patients treated with lecanemab, divided into ARIA‐confirmed (via MRI) and non‐ARIA control groups. Extracellular vesicles (EVs) were isolated and purified from peripheral plasma samples, followed by high‐throughput tRF sequencing. tRF‐19‐J was identified as significantly upregulated in ARIA patients. Its expression levels were validated in an expanded cohort using qRT‐PCR. To explore its functional role, co‐culture models of microglia and endothelial cells were established. Targeted quantitative proteomics (TMT labeling), RNA pull‐down, and RIP‐qPCR were employed to identify tRF‐19‐J binding proteins. Ubiquitination levels of these proteins were assessed using anti‐ubiquitin antibody and immunoprecipitation‐Western blotting (IP‐WB).

**Result:**

Small RNA sequencing revealed a significant upregulation of tRF‐19‐J in ARIA patients, which was confirmed in the validation cohort. Functional assays demonstrated that tRF‐19‐J is secreted by microglia and taken up by endothelial cells, where it binds to connexin 43 (Cx43) in the cytoplasm. This interaction promotes the ubiquitination of Cx43, leading to a reduction in its protein expression levels.

**Conclusion:**

tRF‐19‐J is markedly upregulated in ARIA patients undergoing anti‐Aβ therapy and facilitates the ubiquitin‐mediated degradation of Cx43, suggesting its involvement in BBB permeability disruption. Given its stable expression in peripheral blood, tRF‐19‐J holds potential as a blood‐based biomarker for predicting ARIA in AD patients receiving anti‐Aβ treatment, laying the groundwork for future research into its predictive and therapeutic value.